# Supplementary material for: A Comparative Kidney Transcriptome Analysis of Bicarbonate-Loaded insrr-Null Mice
Source: Curr Issues Mol Biol. 2023 Dec 4;45(12):9709–22. doi: 10.3390/cimb45120606 (PMC10742211; doi:10.3390/cimb45120606)
Supplement: Supplementary file 1 [file cimb-45-00606-s001.zip › cimb-2699240-supplementary-figure.pdf]

current issues in
molecular biology

MDPI

Supplementary Material

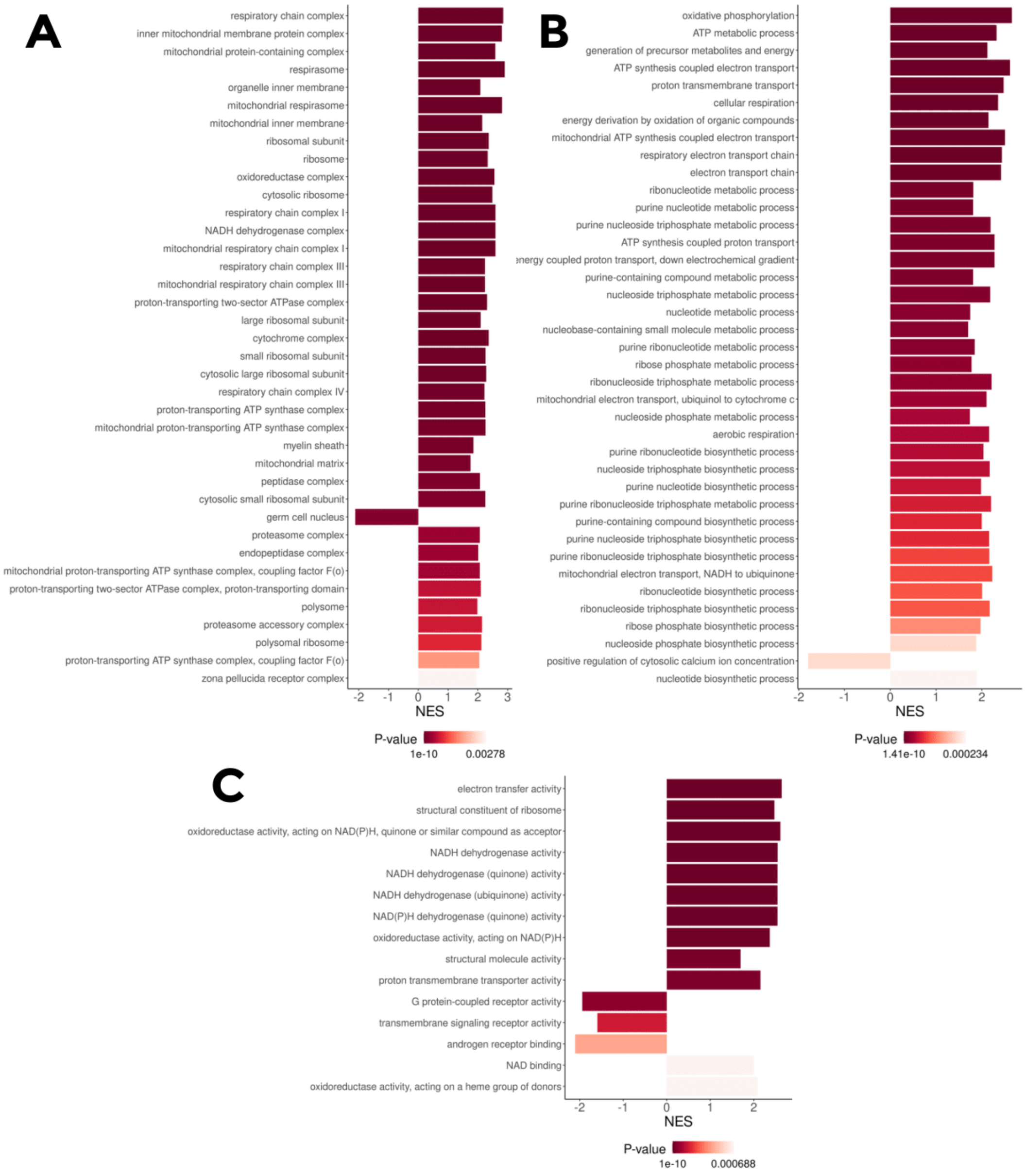

Figure S1. GO terms for which the IRR knockout differential genes under bicarbonate treatment are enriched (fgsea on logFC divided by standard deviation, adjusted $p < 0.05$), NES - normalized enrichment scores (positive - upregulated in knockout, negative - downregulated). A - GO cellular components; B - GO biological process; C - GO molecular function.

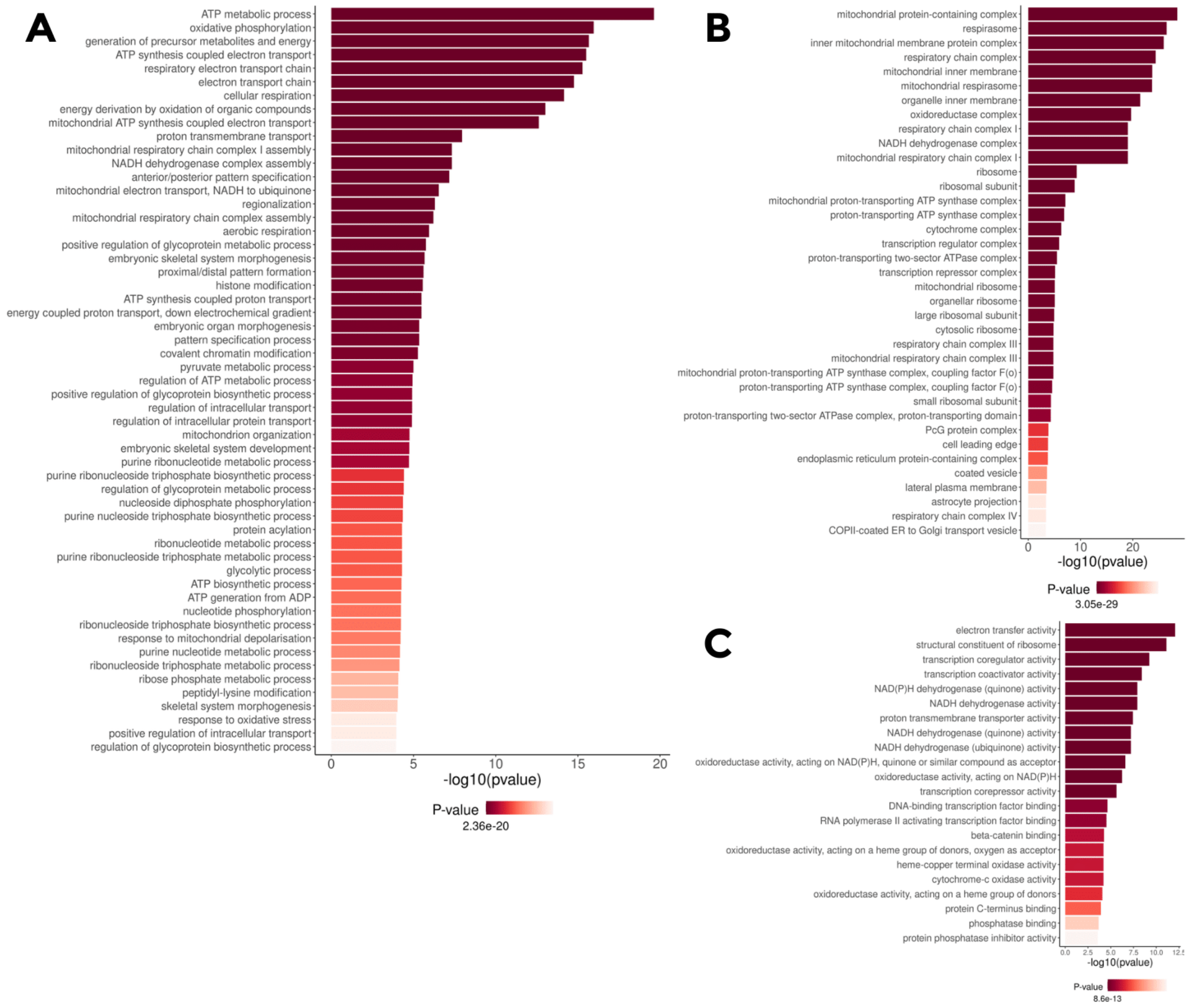

Figure S2. GO terms for which the DE genes from the turquoise WGCNA module which are highly correlated with the module eigengene are enriched (over-representation test, adjusted p < 0.01).

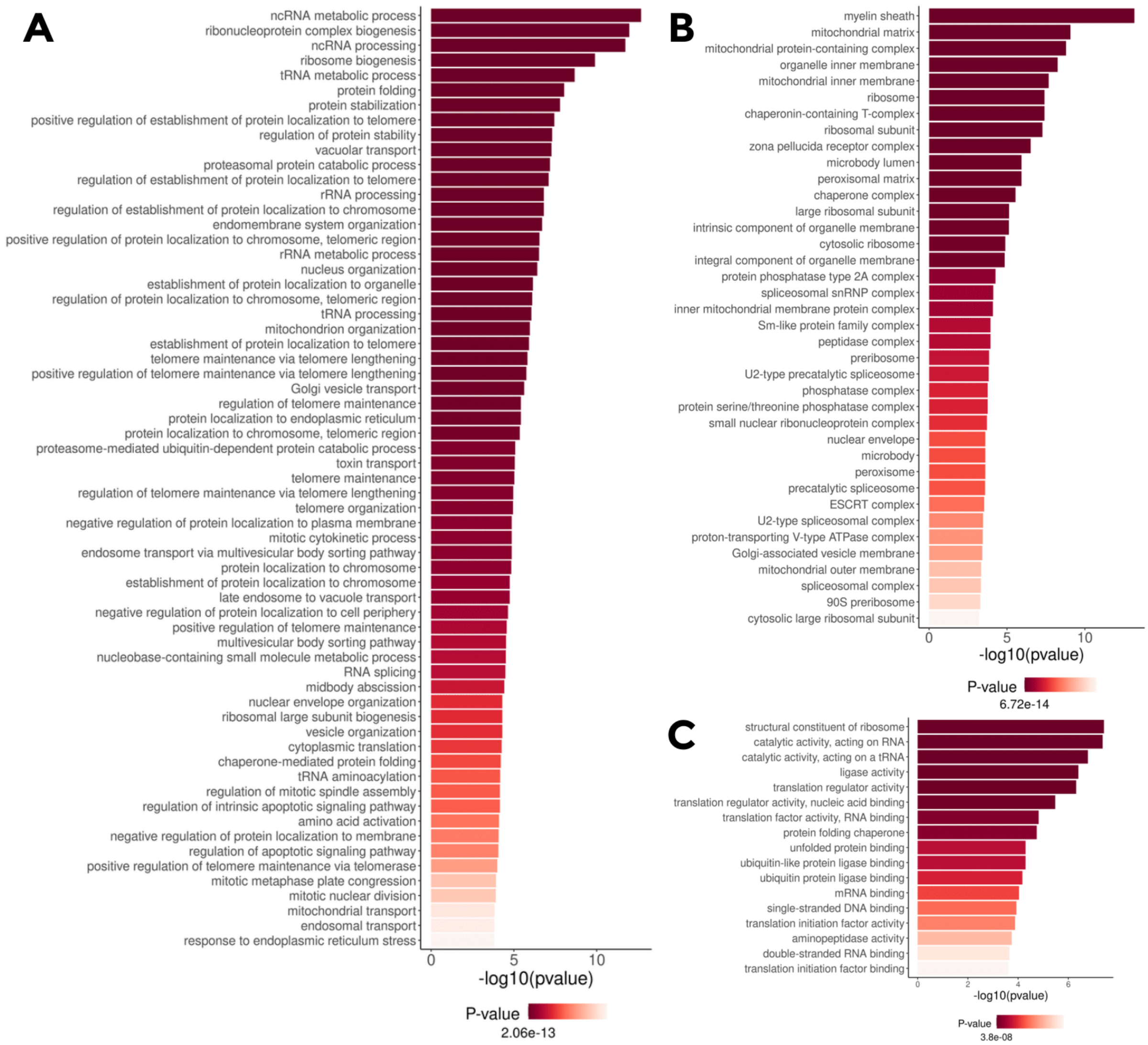

Figure S3. GO terms for which the DE genes from the blue WGCNA module which are highly correlated with the module eigengene are enriched (over-representation test, adjusted p < 0.01).
