# Supplementary material for: A Comparative Kidney Transcriptome Analysis of Bicarbonate-Loaded insrr-Null Mice
Source: Curr Issues Mol Biol. 2023 Dec 4;45(12):9709–22. doi: 10.3390/cimb45120606 (PMC10742211; doi:10.3390/cimb45120606)
Supplement: Supplementary file 1 [file cimb-45-00606-s001.zip › suppemental table S1.pdf]

gene	Log2FoldChange (KO_H2O/WT_H2O)	Log2FoldChange (KO_NaHCO3/WT_NaHCO3)
Mir6236	-2,058263123	-2,280591659
Rny3	2,25686535	1,909055933
Rps3a1	-3,02495289	-1,366796218
Rps7	-1,585694279	-1,773288062
Gapdh	-1,52576309	-1,702721473
Rpl35	1,784358459	1,429682524
Nme2	-1,579963859	-1,548887504
AC161763.1	1,496358239	1,614889571
mt-Nd6	1,471731788	1,641139945
Gm13341	1,646292464	1,463259657
Gm22009	1,639439892	1,412646109
n-R5-8s1	1,831231015	1,228719213
Atn1	1,520501347	1,370618284
Gm4076	1,363485504	1,419551667
S100a11	1,322800524	1,364729957
Pet100	1,35556447	1,308720198
mt-Nd4	1,331661344	1,325597518
Rpl38	-1,145617672	-1,499381338
Fgg	-1,430195634	-1,17688189
mt-Nd2	1,247125958	1,24262486
Rpl29	1,376983997	1,121918675
Atpif1	1,25140621	1,166675352
Tnfsf12	1,087786667	1,330118092
Scarna2	1,292893926	1,047653949
Dynlt1b	-1,222251263	-1,105330061

Supplementary table S1. Top 25 DEGs which changes expression in mouse kidney after *insrr* knockout.
