# Supplementary material for: A Comparative Kidney Transcriptome Analysis of Bicarbonate-Loaded insrr-Null Mice
Source: Curr Issues Mol Biol. 2023 Dec 4;45(12):9709–22. doi: 10.3390/cimb45120606 (PMC10742211; doi:10.3390/cimb45120606)
Supplement: Supplementary file 1 [file cimb-45-00606-s001.zip › suppemental table S2.pdf]

gene	Log2FoldChange (WT_NaHCO3/WT_H2O)	Log2FoldChange (KO_NaHCO3/KO_H2O)
Cptp	0,631934	-0,16362
Nr4a1	0,558604	-0,19521
Lpar2	0,515627	-0,1642
Rcan1	0,503813	-0,1184
Arrdc2	0,489157	-0,08081
Kcnk5	0,417398	-0,08932
Slc52a3	0,277623	-0,34148
Gsr	0,273031	-0,2831
Slc22a12	0,199252	-0,29684
Cbr3	0,187706	-0,40673
Atp5k	0,18106	-0,2838
Atp6v1g3	0,17637	-0,37808
S100a10	0,172713	-0,36741
Pcx	0,134581	-0,3683
Gc	0,131663	-0,46807
Slc51b	0,0979	-0,48568
Svopl	0,096573	-0,45588
Cdc42ep3	-0,09602	0,330925
Nrp2	-0,10993	0,351585
Igfbp3	-0,1592	0,351059
Slc14a2	-0,17895	0,430528
Prelp	-0,18757	0,427061
Wfdc16	-0,25856	0,532557
Tnfsf10	-0,39561	0,15205
Dio1	-0,59044	0,072754

Supplementary table S2. Top 25 DEGs which IRR dependently change expression in mouse kidney under bicarbonate loading.
